# Drinking to toxicity: college students referred for emergency medical evaluation

**DOI:** 10.1186/s13722-016-0059-4

**Published:** 2016-06-08

**Authors:** Sigmund J. Kharasch, David R. McBride, Richard Saitz, Ward P. Myers

**Affiliations:** Division of Pediatric Emergency Medicine, Massachusetts General Hospital, Harvard Medical School, Zero Emerson Place, Suite 3B, Boston, MA 02114 USA; Division of Student Affairs, University of Maryland, College Park, Campus Drive, Building 140, College Park, MD 20742 USA; Department of Community Health Sciences, Boston University School of Public Health, 801 Massachusetts Ave, 4th Floor, Boston, MA 02118 USA; Department of Emergency Medicine, Boston Medical Center, Boston University School of Medicine, One Boston Medical Center Place, Boston, MA 02118 USA

## Abstract

**Background:**

In 2009, a university adopted a policy of emergency department transport of students appearing intoxicated on campus. The objective was to describe the change in ED referrals after policy initiation and describe a group of students at risk for acute alcohol-related morbidity.

**Methods:**

A retrospective cohort of university students during academic years 2007–2011 (September–June) transported to local ED’s was evaluated. Data were compared 2 years prior to initiation of the policy and 3 years after and included total number of ED transports and blood or breath alcohol level.

**Results:**

971 Students were transported to local ED’s. The mean number of yearly transports 2 years prior to policy initiation was 131 and 3 years after was 236 (56 % increase, p < 0.01). 92 % had a blood or breath alcohol level obtained. The mean alcohol level was 193 mg/dL. Twenty percent of students had alcohol levels greater than 250 mg/dL.

**Conclusions:**

Adoption of a university alcohol policy was followed by a significant increase in ED transports of intoxicated students. College students identified as intoxicated frequently drank to toxicity.

## Background

For more than five decades, surveys have documented the excessive and pervasive use of alcohol on U.S. college campuses [1–3]. The Office of the Surgeon General has characterized high-risk drinking among college students as a major public health problem [4] and nearly one-third meet the diagnostic criteria for alcohol use disorder [5]. It is estimated that 1700 college students die each year from alcohol related injuries [6].

Studies of alcohol use among college students have relied predominantly on self-report data, and objective measures of alcohol consumption among this group have infrequently been verified. The Harvard School of Public Health College Alcohol Surveys (CAS) conducted four times between 1993 and 2001 reported the sustained prevalence (40–45 %) of heavy episodic (“binge”) drinking among college students nationwide and described the increased prevalence of impaired cognition, diminished academic performance, alcohol-related injuries, engagement in vandalism, risky sexual activity, and drinking while driving among students who engaged in binge drinking [7]. Other national surveys have confirmed a similar prevalence of binge drinking among college students [8, 9].

In 2004, the National Institute of Alcohol Abuse and Alcoholism (NIAAA) defined a binge “as a pattern of drinking alcohol that brings blood alcohol concentration to 0.08 g percent or above and corresponds to the 4+/5+ level of consumption for females and males within about a 2 h period” [10]. However, studies have found that drinking at these levels in a college population frequently results in blood alcohol concentrations much lower than the 0.08 threshold [11]. Additionally, students often, poorly estimate the actual volume of a drink [12], adding further confusion as to the true nature of college drinking patterns and behaviors.

Blood alcohol concentration (BAC) or breath alcohol concentration (BrAC) to assess intoxication levels in a collegiate environment is an important adjunct to self-report surveys or interviews. Previous studies comparing BAC to BrAC have demonstrated near equivalency [13, 14]. Emergency departments (EDs) are uniquely situated to collect such objective data in order to elucidate the level of college drinking behavior. In previous ED studies of college students with alcohol intoxication at Vanderbilt University and the University of Virginia, the epidemiology of alcohol–related problems and intoxication among college students were described. At Vanderbilt University, 101 (16.4 %) of 616 undergraduate ED encounters over one academic year were alcohol related and 28 % presented with clinical or laboratory findings of severe alcohol intoxication. Four percent of students were hospitalized for medical complications and one student died of head trauma. Overall, it was estimated that 1 of every 15 undergraduates seen in the campus ED had alcohol-related problems. At the University of Virginia, 193 (13 %) of 1529 ED encounters over two academic years were alcohol related. Thirty-four percent presented with clinical or laboratory findings of severe alcohol intoxication and 53 % had associated trauma related to alcohol. Five percent of students were hospitalized for trauma or ventilator support for alcohol poisoning. However, alcohol levels were measured in only 21 and 16 % of students respectively, thereby limiting the scope of objective data collected [15, 16].

Several prevention practices and policies exist on college campuses to address alcohol-related problems. Binge drinking prevention initiatives at U.S. colleges and universities range from alcohol education, prohibitions on alcohol access, alcohol-free campus housing and activities, and restrictions on alcohol advertising [17–19]. However, few individual policies have been evaluated for their effectiveness [20] and implementation of specific policies varies by size and location of schools as well as by college administrators’ perception of the importance of alcohol use as a problem on campuses [21]. Recently, two universities have implemented “arrest-first” policies for violations of alcohol laws [22]. Strict enforcement of existing policies may be associated with reductions of alcohol use among college students [23].

Additional research has focused on the student’s role in assisting other students with alcohol intoxication or poisoning to try to avoid students not seeking help when needed, potentially avoiding preventable deaths. In a study of students at a Midwest university, 58 % of students indicated they had helped another student with symptoms of alcohol poisoning but did not seek outside help under such circumstances. Students more reluctant to seek help did not do so primarily due to their inability to distinguish symptoms of alcohol poisoning and their perception that help was not needed [24]. Increasingly, universities have adopted Good Samaritan or medical amnesty policies (MAP) to eliminate or reduce campus judicial consequences and encourage college students to seek help in cases of alcohol poisoning. At Cornell University, calls to emergency medical services (EMS) for assistance with intoxicated students increased after initiation of MAP [25]. Combining alcohol-poisoning education with MAP has been found to have the greatest impact on help-seeking behavior of students [26].

Responding in part to highly publicized alcohol related tragedies in Massachusetts as well as ongoing feedback from our ED to University Student Health Services regarding alcohol levels, intoxication and poisoning among its student population, a large, local university in Boston instituted a policy of university police notification in 2009 with subsequent ED transport by EMS of college students on campus identified with presumed alcohol intoxication. For several years prior to implementation of this policy, it had been the practice of the University to intermittently transport intoxicated students to ED’s. The purpose of the policy was to ensure the safety and well being of students and all members of the university community by requiring EMS transports to local ED’s for medical evaluation of intoxicated students who might otherwise suffer serious health consequences or harm others. The purpose of this study was to evaluate the change in ED referral patterns from before to after adoption of a university-wide policy and to describe a large group of university students at risk for acute alcohol-related morbidity. It was hypothesized that adoption of such a policy would be followed by an increase in ED transports and that many of these identified students consume alcohol at potentially dangerous alcohol levels, as measured in blood or breath.

## Methods

A retrospective cohort of university students transported to local ED’s from academic years 2007–2011 was evaluated. Undergraduate students during the specified academic years were included in the study. The university health center and police maintain a list of all students brought to ED’s with intoxication (EMS transports and ED contact person) for clinical follow-up purposes. Prior to the policy initiation in academic year 2009, the university notified (through group departmental meetings and emails) campus housing personnel, security, and resident advisors of the new protocol. Students exhibiting signs or symptoms of acute alcohol intoxication or poisoning on campus including ataxia, slurred speech, vomiting, disorientation, or alterations in consciousness by university personnel or calls for help by other students, had the university police called and subsequent EMS transport to local emergency rooms. Demographic data (name, date of birth, date of visit) were sent from the university health center to the Boston Medical Center (BMC) data warehouse, and de-identified clinical data were provided to the study investigators. Data were compared 2 years prior to initiation of the policy and 3 years after. Total number of ED transports and blood or breath alcohol levels were compared for the two time periods using a Welch *t* test, assuming unequal variance. Breath alcohol levels were obtained on patients presenting with clinical intoxication. For patients unable to provide a breath alcohol level (e.g., vomiting, diminished level of consciousness) a blood alcohol level was obtained. The distribution of alcohol levels was tested and confirmed to be normal using the Shapiro–Wilks test. Gender, age, length of stay, disposition and associated diagnosis were tabulated for the BMC group. Proportion of visits for males and females were compared using the exact binomial test. Alcohol levels for males and females were compared using a Welch t-test, assuming unequal variance. The Boston University Institutional Review Board approved the study protocol. Statistical analyses were performed in R [27].

## Results

**Fig. 1:**
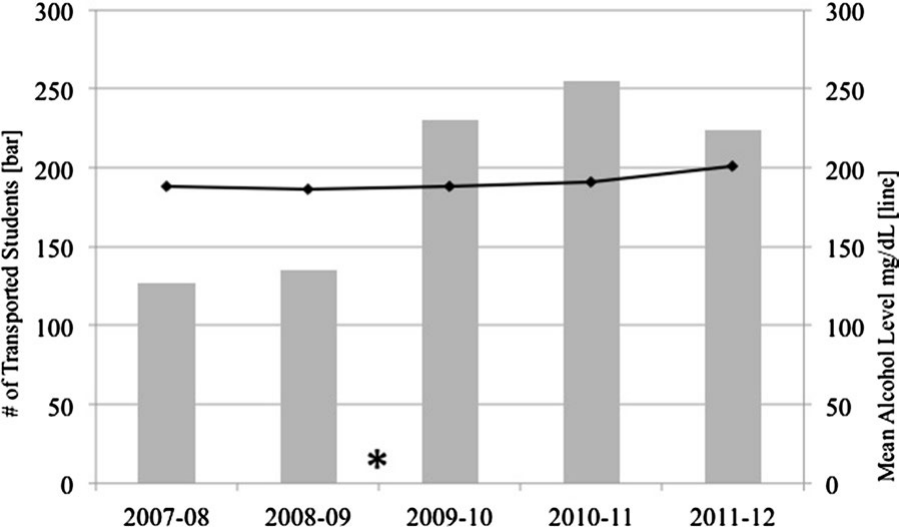
Yearly number of transported students and mean alcohol levels. Policy initiation noted by *asterisk*. Change in transported students pre/post policy is significant at p < 0.01 level

Between academic years 2007 and 2011, 971 students were transported to ED’s for presumed alcohol intoxication. The mean number of yearly transports 2 years prior to policy initiation was 131 and 3 years after was 236 (Fig. 1, 56 % increase, p < 0.01). Despite a nearly two-fold increased in the number of students transported after policy initiation, the mean alcohol level did not significantly change (Fig. 1). During the five academic years, 679 students (70 %) were brought to the BMC ED. Of the BMC group, 55 % were female. The mean age was 19 years with 84 % being below the legal drinking age. The mean alcohol level was 193 and mean length of stay was 252.5 min. Males had a significantly higher mean alcohol level (199 vs. 188 mg/dL, p < 0.05), although this difference may not be clinically significant. Nine (1.3 %) students were hospitalized for trauma or medical conditions (including five closed head injuries, one diabetic ketoacidosis, one vomiting/hypoglycemia, one incidental brain mass and one psychosis) and were eventually discharged. The mean alcohol level of admitted patients (236 mg/dL) was significantly greater than was the mean level for those not admitted (193 mg/dL) (p < 0.01). Nine percent had at least two repeat visits for alcohol intoxication within the study period.

**Fig. 2:**
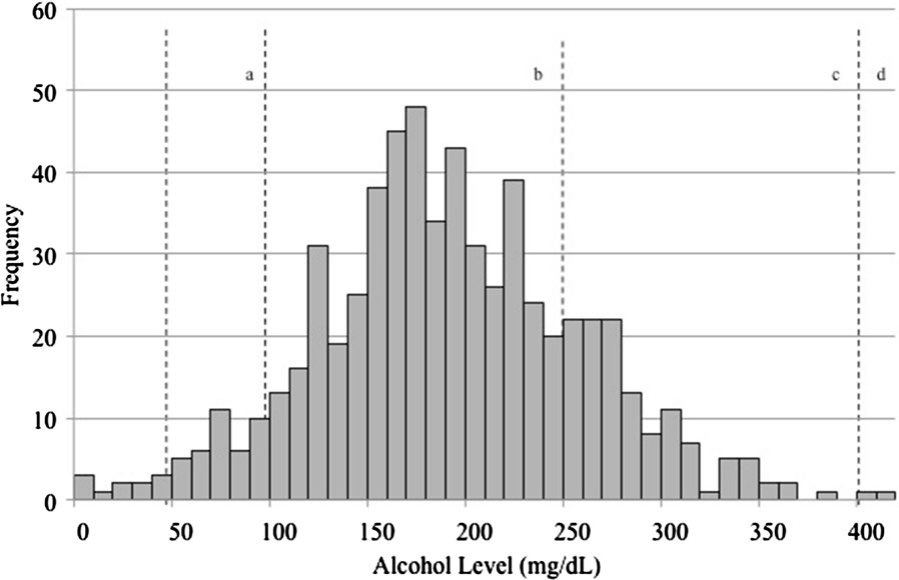
Histogram of alcohol levels. *Notes*: levels typically associated with following symptoms. *a*—decreased judgment and coordination. *b*—ataxia slurred speech, vomiting. *c*—stupor or coma, incontinence. *d*—loss of protective airway reflexes, hypothermia, death

Six hundred and twenty-six (92 %) of students had a BAC or BrAC level performed. Ninety-five percent had an alcohol level greater than 80 mg/dL. Seventy-three percent had a level between 100 and 250 mg/dL and 20 % had a level between 250 and 400 mg/dL (Fig. 2). Students with alcohol levels >250 mg/dL were significantly more likely to be male (57 vs. 41 %, p < 0.01), older (19.2 vs. 18.9 years, p < 0.05), have a longer length of stay (358 vs. 232 min, p < 0.01), more likely to be restrained (9 vs. 1 %, p < 0.001), and more likely to have an imaging study (10 vs. 3 %, p < 0.01).

## Comment

Studies of alcohol use among college students have relied predominantly on self-report survey data and utilizing objective measures of alcohol consumption has been identified as an important research priority [28].

To our knowledge, the current study is the first comprehensive use of blood or breath alcohol screening among college students and provides an important window into alcohol consumption behaviors. Almost all university students transported to the emergency room in our data had a blood or breath alcohol level measured. The mean alcohol level was high and almost all (95 %) had alcohol levels greater than the NIAAA definition (80 mg/dL) for binge drinking and 70 % had alcohol concentrations two times (160 mg/dL) this level. In a previous study focusing on extreme drinking practices in college freshman, 50 % of males and 24 % of females categorized as binge drinkers drank at levels at least twice the binge threshold (10 + males, 8 + females) [29]. Students that engage in extreme drinking practices have a significantly increased risk of alcohol related injuries compared to those that drink at the 4+/5+ threshold [30].

Most students in our study had between 100 and 250 mg/dL, levels where clinical effects of cerebellar and vestibular dysfunction (ataxia, slurred speech, diplopia, nystagmus), confusion, nausea and vomiting and stupor may occur; a substantial minority had alcohol levels between 250 and 400 mg/dL, levels that have been associated with respiratory depression, stupor, coma, and death [31].

Binge drinking among 18–22 year olds not enrolled in college decreased significantly though by a small absolute amount from 2002 (39 %) to 2010 (36 %). However, among college students 18–22 years of age, binge drinking has remained relatively stable from 2002 (44 %) to 2010 (42.2 %) [32]. While adoption of the policy in the current study was followed by a 56 % increase in ED transports, we cannot infer a cause-effect relationship due to the lack of a comparison group. For example, at the university evaluated in this study, alcohol education and assessment programs for students (BASICS, e-CHUG) and other efforts informing students of the dangers of heavy alcohol consumption occurred simultaneously and following policy adoption. While it was beyond the scope of the current study to assess the impact of such programs on alcohol transports, increased student awareness as to the dangers of alcohol intoxication or poisoning could have influenced calls for assistance for such students and hence EMS transports.

Nevertheless, the finding that blood or breath alcohol concentrations were not significantly different before or after policy initiation highlights the potentially effective practice of behavioral identification of intoxicated students by university personnel; presumably many of such students in prior years would not have presented for care and could have suffered consequences. While adoption of such a policy is not an alcohol prevention practice per se, it is perhaps better viewed as an alcohol safety policy. The fact that 20 % of students had alcohol levels greater than 250 mg/dL, underscores the toxic level of drinking behaviors among college students and subsequent potential for disastrous health consequences. While most students were eventually discharged, they can be safely monitored and treated in an ED environment for potential airway complications (respiratory depression, aspiration), trauma, on-going fluid losses and other complications of acute alcohol intoxication.

Our study had important strengths, primarily in the use of objective measures to verify the levels of drinking behavior in a college population that has not been previously described. Ninety-two percent of students in the current study had blood or breath alcohol level obtained which provides additional new and important information in our understanding of alcohol use among college students. The toxic level of drinking described in our study highlights the importance of studies of campus policies that focus on early identification and medical evaluation of students that engage in high-risk drinking in order to avoid dire health consequences.

## Limitations

Our study had several important limitations. First, although most were, not all students were brought to the BMC ED. However, we have no reason to believe that students brought to other EDs in Boston were clinically different than the BMC group. Second, the experience described here is limited to only one university and may not be generalizable to others. Additionally, other efforts at the university as discussed above may have influenced the increase in EMS transports of intoxicated students. Lastly, the students transported to EDs in Boston may represent only the minority of students that were identified as engaging in extreme drinking practices on a college campus and certainly raises concerns regarding selection bias. Nevertheless, we believe that the current study provides objective criteria to better understand the potentially dangerous aspect of college students drinking practices.

## Conclusions

Our study findings demonstrate that college students identified as intoxicated and brought to an emergency department frequently drink to toxicity as measured by blood or breath alcohol levels. Medical amnesty policies, arrest first policies and alcohol poisoning education are potential areas of study and policy development that may impact the safety and well being of college students. Such studies should go beyond single universities, involve quasi-experimental designs, and consider other policies and practices implemented.

Despite the limitations in the current study, we do believe that these data are sufficient for universities to consider such approaches as part of comprehensive alcohol strategies, perhaps evaluating such policies as they are put in place. Adoption of a university alcohol policy that identifies intoxicated students with subsequent EMS transport to an ED, may provide an important safety measure to reduce alcohol morbidity and mortality among college students.
